# Molecular epidemiological and antimicrobial-resistant mechanisms analysis of prolonged *Neisseria gonorrhoeae* collection between 1971 and 2005 in Japan

**DOI:** 10.1093/jacamr/dlae040

**Published:** 2024-03-12

**Authors:** Narito Kagawa, Kotaro Aoki, Kohji Komori, Yoshikazu Ishii, Ken Shimuta, Makoto Ohnishi, Kazuhiro Tateda

**Affiliations:** Department of Microbiology and Infection Control and Prevention, Toho University Graduate School of Medicine, 5-21-16 Omori-nishi, Ota-ku, Tokyo 143-8540, Japan; Department of Microbiology, School of Life and Environmental Science, Azabu University, Kanagawa, Japan; Department of Microbiology and Infectious Diseases, Toho University School of Medicine, Tokyo, Japan; Department of Microbiology and Infection Control and Prevention, Toho University Graduate School of Medicine, 5-21-16 Omori-nishi, Ota-ku, Tokyo 143-8540, Japan; Department of Microbiology and Infection Control and Prevention, Toho University Graduate School of Medicine, 5-21-16 Omori-nishi, Ota-ku, Tokyo 143-8540, Japan; Department of Microbiology and Infectious Diseases, Toho University School of Medicine, Tokyo, Japan; Department of Bacteriology I, National Institute of Infectious Diseases, Tokyo, Japan; Antimicrobial Resistance Research Center, National Institute of Infectious Diseases, Tokyo, Japan; Department of Bacteriology I, National Institute of Infectious Diseases, Tokyo, Japan; Department of Microbiology and Infection Control and Prevention, Toho University Graduate School of Medicine, 5-21-16 Omori-nishi, Ota-ku, Tokyo 143-8540, Japan; Department of Microbiology and Infectious Diseases, Toho University School of Medicine, Tokyo, Japan

## Abstract

**Objectives:**

As antimicrobial-resistant (AMR) *Neisseria gonorrhoeae* strains have emerged, humans have adjusted the antimicrobials used to treat infections. We identified shifts in the *N. gonorrhoeae* population and the determinants of AMR strains isolated during the recurring emergence of resistant strains and changes in antimicrobial therapies.

**Methods:**

We examined 243 *N. gonorrhoeae* strains corrected at the Kanagawa Prefectural Institute of Public Health, Kanagawa, Japan, these isolated in 1971–2005. We performed multilocus sequence typing and AMR determinants (*penA*, *mtrR*, *porB*, *ponA*, 23S rRNA, *gyrA* and *parC*) mainly using high-throughput genotyping methods together with draft whole-genome sequencing on the MiSeq (Illumina) platform.

**Results:**

All 243 strains were divided into 83 STs. ST1901 (*n* = 17) was predominant and first identified after 2001. Forty-two STs were isolated in the 1970s, 34 in the 1980s, 22 in the 1990s and 13 in the 2000s, indicating a decline in ST diversity over these decades. Among the 29 strains isolated after 2001, 28 were highly resistant to ciprofloxacin (MIC ≥ 8 mg/L) with two or more amino-acid substitutions in quinolone-resistance-determining regions. Seven strains belonging to ST7363 (*n* = 3), ST1596 (*n* = 3) and ST1901 (*n* = 1) were not susceptible to cefixime, and six strains carried *penA* alleles with mosaic-like penicillin-binding protein 2 (PBP2; *penA* 10.001 and 10.016) or PBP2 substitutions A501V and A517G.

**Conclusions:**

We observed a significant reduction in the diversity of *N. gonorrhoeae* over 35 years in Japan. Since 2001, ST1901, which is resistant to ciprofloxacin, has superseded previous strains, becoming the predominant ST population.

## Introduction

The spread of antimicrobial resistance (AMR) in *Neisseria gonorrhoeae* represents a significant public health concern.^1,2^ Historically, when resistant strains of *N. gonorrhoeae* have emerged, the primary antimicrobial agents used were altered to accommodate the changes.^3^ To devise effective treatments for *N. gonorrhoeae* infections in the future, a thorough understanding of both the historical emergence of AMR and the molecular biology of the resistance mechanisms is essential.

The history of treating *N. gonorrhoeae* infections is marked by the recurrent emergence of AMR strains, requiring changes in the primary antimicrobial agent used to treat them. Although penicillin-resistant *N. gonorrhoeae* strains have been reported since 1946, penicillin remained the treatment of choice for gonorrhoea until the early 1980s.^3–5^ In 1976, penicillinase-producing *N. gonorrhoeae* emerged and spread rapidly internationally.^6–8^ Subsequent to this, by the mid- to late-1980s, fluoroquinolones had become the preferred treatment for penicillin-resistant *N. gonorrhoeae* infections. However, a noticeable increase in ciprofloxacin-resistant strains in Japan began after 1992.^9–11^ This resistance to fluoroquinolones in *N. gonorrhoeae* predominantly arises from amino-acid substitutions within the quinolone-resistance-determining regions (QRDRs) of DNA gyrase subunit A (GyrA) and topoisomerase IV subunit A (ParC).^12^ Oral expanded-spectrum cephalosporins (ESCs), such as cefixime, have been primarily used since the early 1990s. However, reports of emerging cefixime-resistant (CFM-R) *N. gonorrhoeae* were published in Japan in 1995.^13^ The frequency of CFM-R isolates increased after 2000.^14,15^ The CFM-R determinants entail reduced affinity for ESCs due to amino-acid substitutions in penicillin-binding protein 2 (PBP2) or an acquired mosaic-like mutation in PBP2 (encoded by the *penA* allele with the mosaic-like mutation: *penA*^mosaic^). Consequently, the first-line treatment for *N. gonorrhoeae* infection has been changed to ceftriaxone or spectinomycin in Japan.^3^ In 2009, a high-level ceftriaxone-resistant (MIC = 2 mg/L) *N. gonorrhoeae* strain, H041, part of ST7363 (designated ‘WHO X’ of the WHO reference strains), was identified in Kyoto, Japan.^16^ Ceftriaxone-resistant strains (MIC = 0.5 mg/L) were subsequently isolated in 2014 (GU140106) and 2015 (FC428), belonging to ST7363 and ST1903, respectively.^17–19^ In 2010, a strain known as F89 (ceftriaxone MIC = 1–2 mg/L, and belonging to ST1901) was reported in France and Spain.^20,21^ These strains had acquired the *penA*^mosaic^ mutation, including key changes in PBP2, contributing to their ceftriaxone resistance.^16,22^

Available information on STs of *N. gonorrhoeae* strains isolated before 2000 is limited, because the multilocus sequence typing (MLST) schema for *Neisseria* species only became available from 1998 onwards. Furthermore, there are few data on the AMR determinants, such as *penA* alleles or QRDRs, primarily because of the prohibitively high sequencing costs associated with MLST and its analysis at that time.^23,24^ Consequently, a comprehensive genotypic study and analysis of AMR determinants of the strains isolated during the period preceding and concurrent with the use of fluoroquinolones and ESCs could provide invaluable insights into the influence of therapeutic agents on the gonococcal population.

In this study, we genotyped and analysed the AMR determinants prevalent in the AMR *N. gonorrhoeae* strains isolated between 1971 and 2005. Our purpose was to unravel the intricacies of the gonococcal population, the lineages of AMR strains and the evolution of the resistance mechanisms that have culminated in the emergence of AMR *N. gonorrhoeae*. These bacteria continue to pose significant challenges today.

## Methods

### Used strains

**Figure 1.**
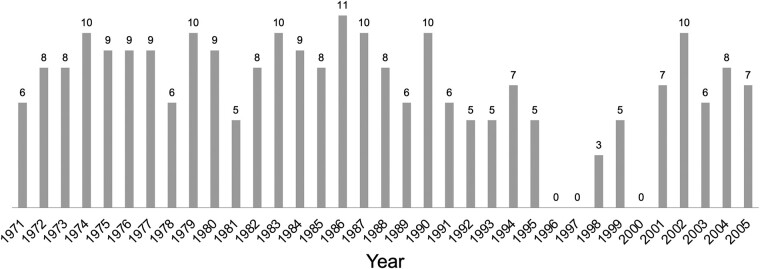
Year of isolation and distribution of 243 strains of *Neisseria gonorrhoeae*. We examined up to 12 strains each year. No strains were available in 1996, 1997 or 2000.

We used 243 *N. gonorrhoeae* strains randomly chosen from a strain collection predominantly isolated in Tokyo and Kanagawa Prefecture. This collection, located in Kanagawa Prefecture, which is adjacent to the southern region of Tokyo, Japan, includes strains from the period 1971–2005 (Figure 1). The criteria for strain selection included a maximum of 12 strains per year without reference to antimicrobial susceptibility data. The collection is stored as gelatin discs at the Kanagawa Prefectural Institute of Public Health. We recovered them, stored them in glycerol stocks at −80°C and used them in this study.

### Antimicrobial susceptibility testing

We measured the antimicrobial susceptibility of all the strains in this study. MICs were determined with the agar dilution method, according to the CLSI M07-ED11 guidelines.^25^ MICs were interpreted according to the clinical breakpoint of CLSI M100-ED33.^26^ ‘Non-susceptible’ was defined as >0.25 mg/L for cefixime and ceftriaxone, and >1 mg/L for azithromycin. The MICs of the following six antimicrobial agents were measured: penicillin (concentration range 0.03–64 mg/L), cefixime (0.015–1 mg/L), ceftriaxone (0.015–1 mg/L), spectinomycin (0.5–256 mg/L), ciprofloxacin (0.03–64 mg/L) and azithromycin (0.03–64 mg/L). *N. gonorrhoeae* ATCC 49226 was used as the reference strain in antimicrobial susceptibility testing to maintain quality control.

### Molecular epidemiological characterization with short-read sequencing using the Illumina platform

We sequenced seven MLST alleles (*abcZ*, *adk*, *aroE*, *fumC*, *gdh*, *pdhC* and *pgm*) and seven alleles of the *N. gonorrhoeae* Sequence Typing for Antimicrobial Resistance (NG-STAR) molecular typing scheme (*penA*, *mtrR*, *porB*, *ponA*, 23S rRNA, *gyrA* and *parC*) with a high-throughput typing method for *N. gonorrhoeae,* according to a previous report.^27^ The genomic sequence data for 14 WHO reference strains were used to validate the high-throughput typing method. Other AMR determinants were analysed with ResFinder4.1.^28^

In brief, multiplex PCR was conducted for target amplification, followed by indexing PCR for sequencing with the MiSeq (Illumina, San Diego, CA, USA) platform. Alleles were identified or substitutions detected with the PubMLST and NG-STAR databases as references,^29,30^ If the high-throughput typing method was unsuccessful for some samples, we performed a draft whole-genome sequencing (WGS) analysis with the Tagmentation method, exclusively for those strains. We prepared DNA libraries with the Illumina DNA Prep, (M) Tagmentation kit (Illumina) and sequenced 300 bp × 2 paired-end reads with MiSeq. Draft genome contigs were generated with *de novo* assembly using SPAdes v.3.15.3.^31^

### Gene annotation and alignment analysis

The nucleotide sequences were annotated with the DNA Data Bank of Japan Fast Annotation and Submission Tool.^32^ Alignment of contigs obtained through *de novo* assembly to a reference genome sequencing was performed with Multiple Alignment of Conserved Genomic Sequence with Rearrangements.^33^

## Supplementary Material

dlae040_Supplementary_Data

## Figures and Tables

**Figure 2.**
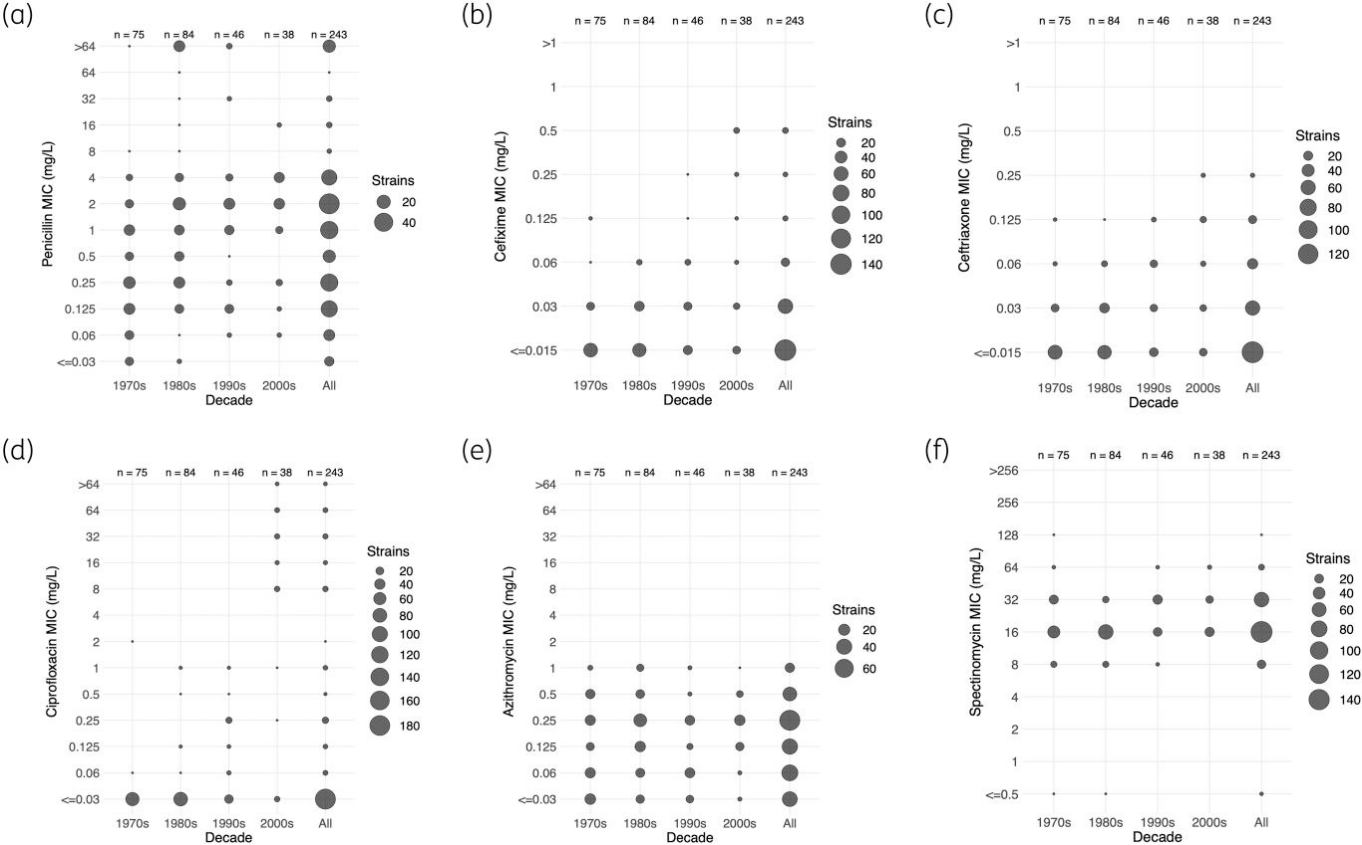
Antimicrobial susceptibility of 243 *Neisseria gonorrhoeae* strains. Relationship between MIC and the isolation decade is shown in a bubble plot. (a) penicillin; (b) cefixime; (c) ceftriaxone; (d) ciprofloxacin; (e) azithromycin and (f) spectinomycin.

**Figure 3.**
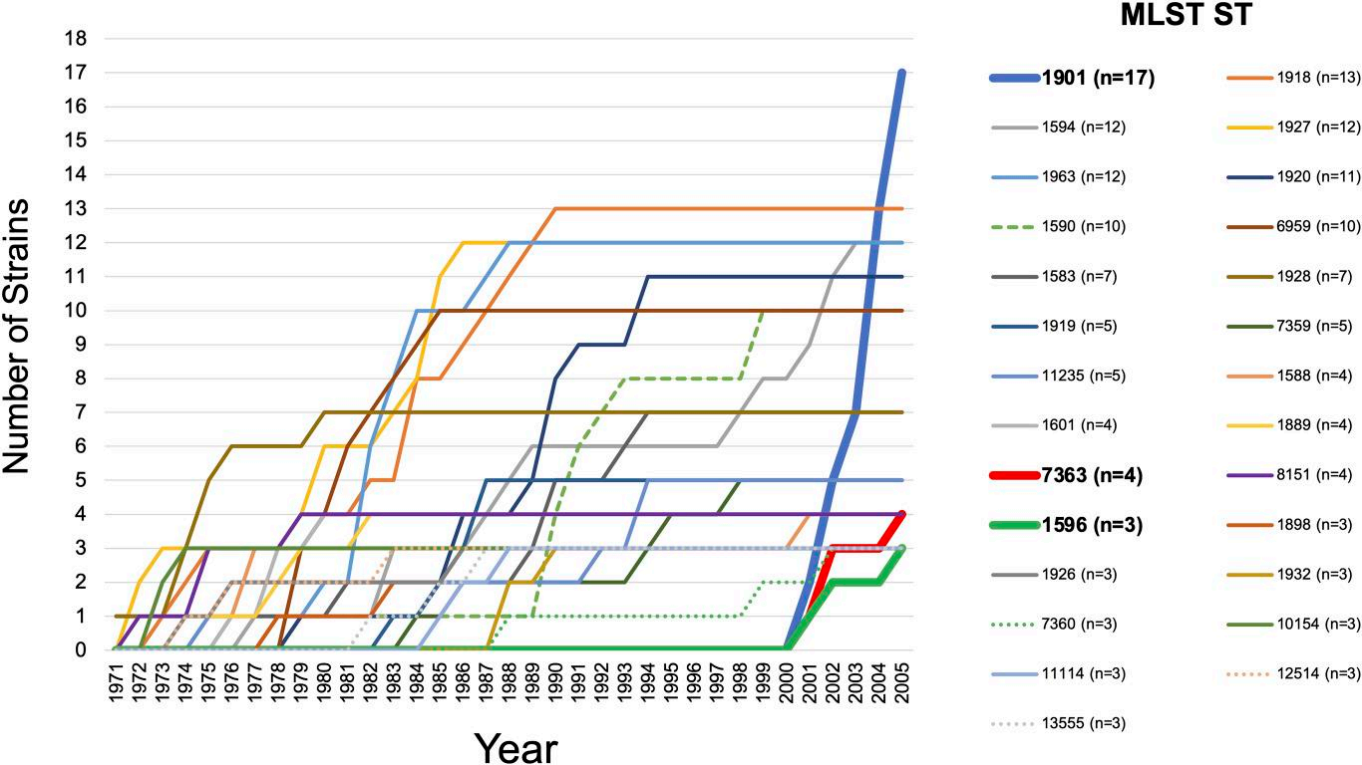
Cumulative line graph of isolation years by MLST-ST. STs with three or more strains in this study are shown. Bold font indicates STs that were isolated for the first time since 2001.

**Figure 4.**
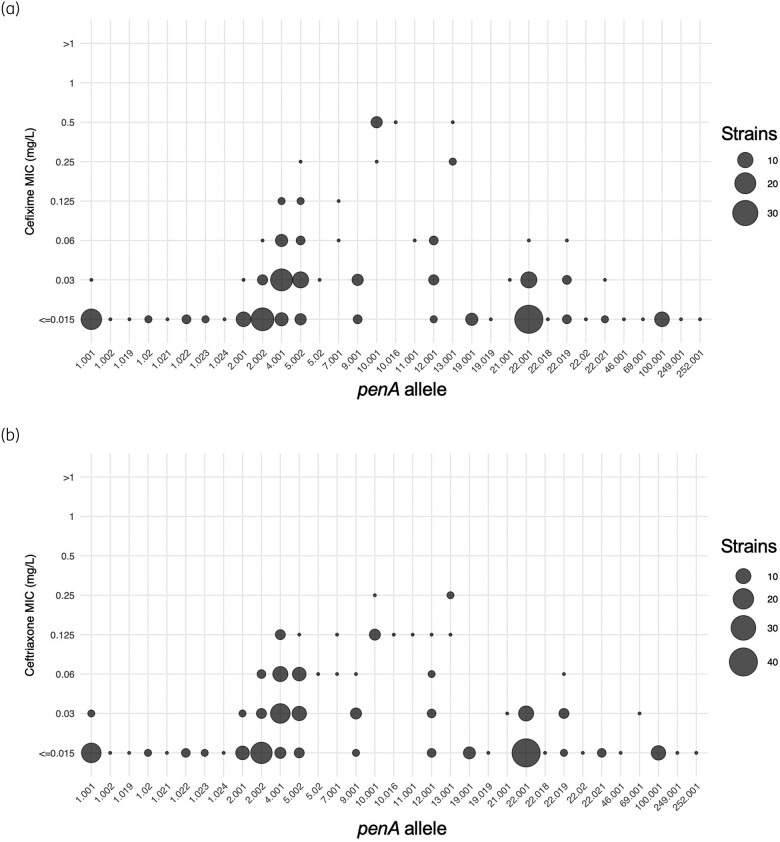
Relationships between cefixime or ceftriaxone and *penA* mutation. (a) Cefixime MIC for each strain carrying the *penA* allele and (b) ceftriaxone MIC for each strain carrying the *penA* allele.

**Figure 5.**
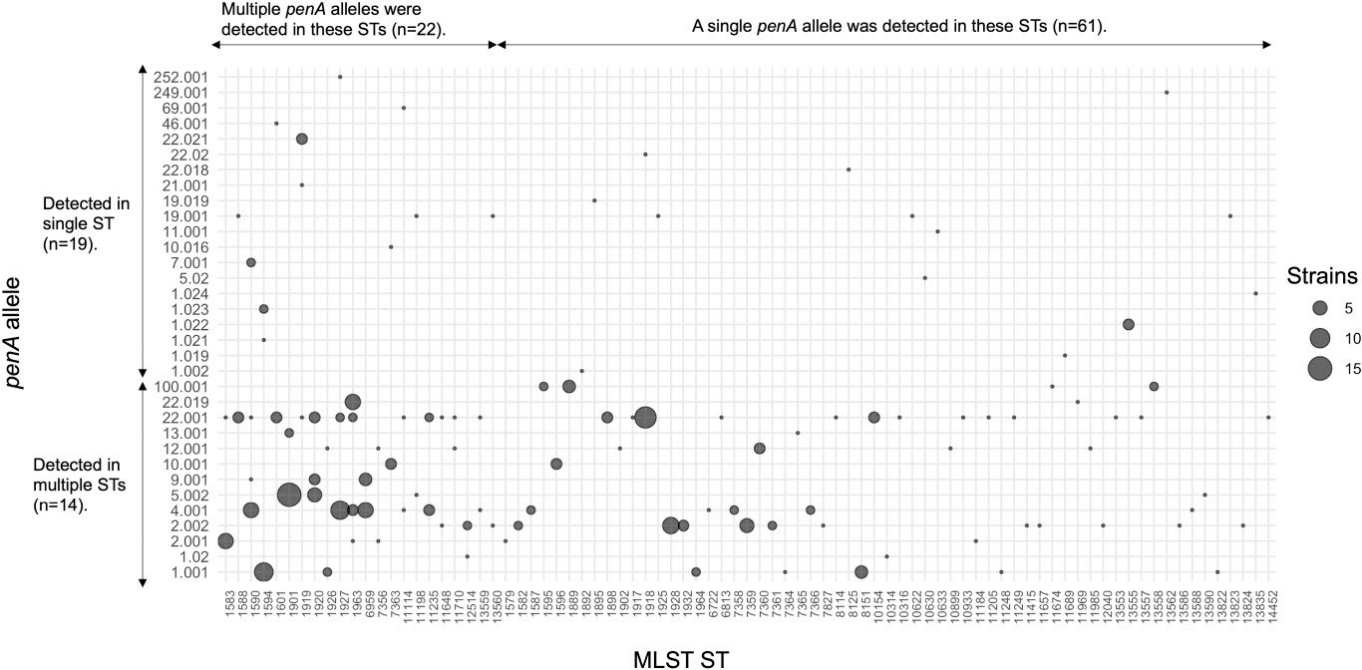
Strains grouped by MLST and the *penA* alleles carried. The relationship between ST and the *penA* allele carried is shown in a bubble plot.

**Figure 6.**
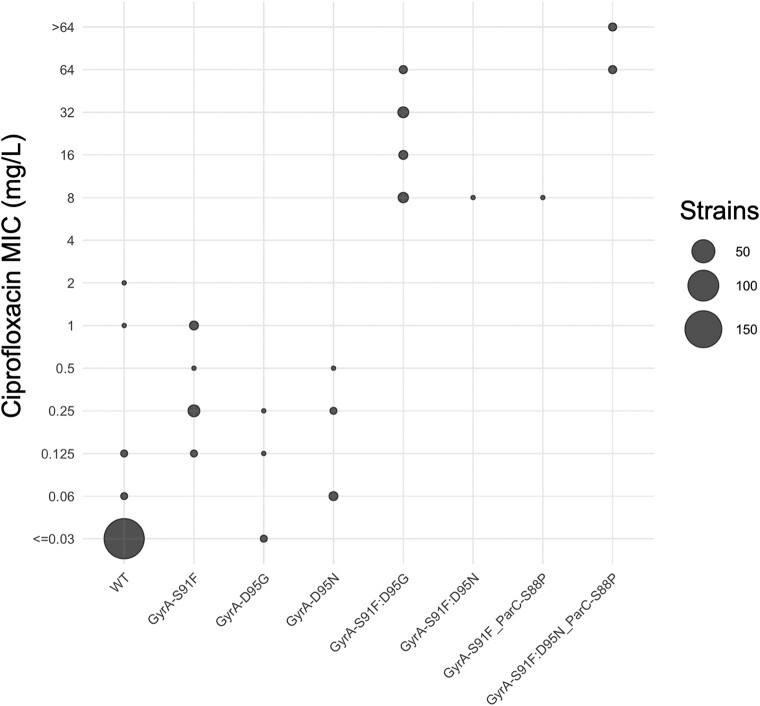
Relationships between QRDR mutations and susceptibility to ciprofloxacin. GyrA-S91F, serine at position 91 of GyrA is replaced by phenylalanine; GyrA-D95G, aspartic acid at position 95 of GyrA is replaced by glycine; ParC-S88P, serine at position 88 of ParC is replaced by proline.

**Figure 7.**
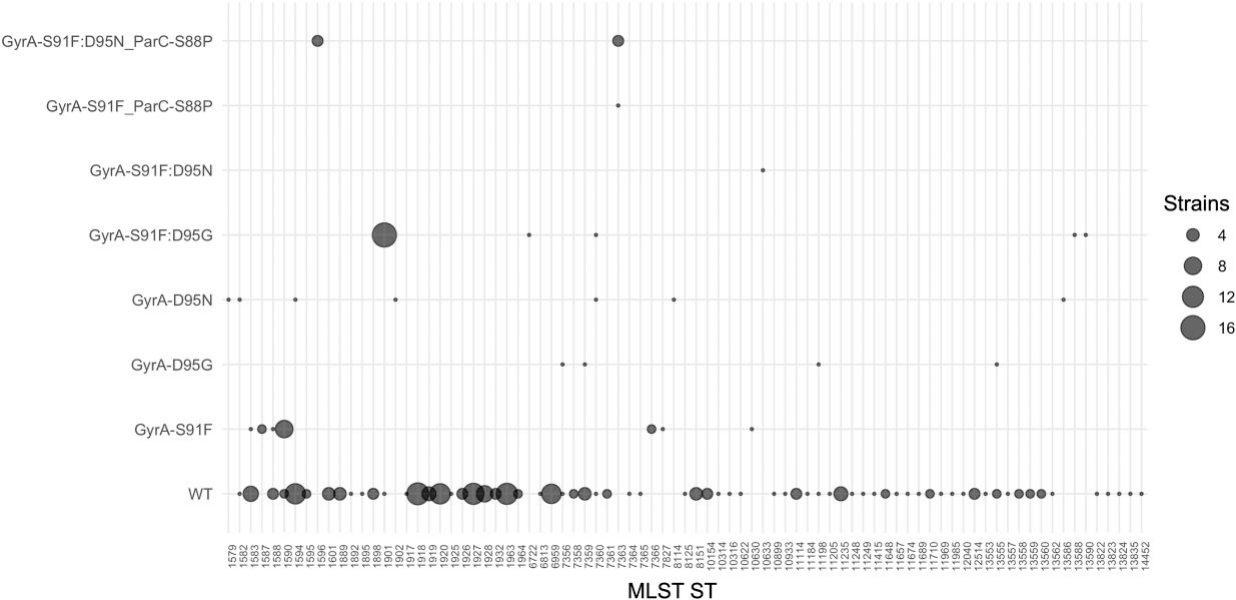
Relationship between QRDR mutations and associated MLST. GyrA-S91F, serine at position 91 of GyrA is replaced by phenylalanine; GyrA-D95G, aspartic acid at position 95 of GyrA is replaced by glycine; ParC-S88P, serine at position 88 of ParC is replaced by proline.

**Figure 8.**
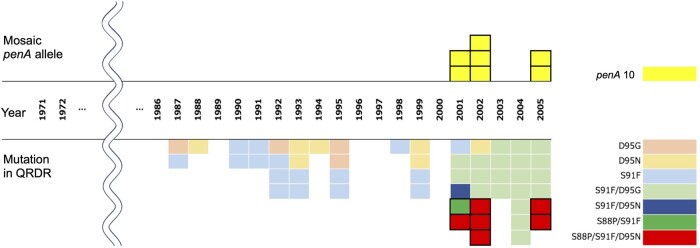
Time line of emerging strains with a mutation in QRDR and/or carrying *penA* 10. Lower section shows strains with mutations in the QRDR, and the upper section shows strains carrying *penA* 10. Bold squares indicate strains with both QRDR mutations and *penA* 10.

## Data Availability

The MiSeq sequencing reads have been deposited in GenBank under BioProject accession number PRJNA992923. The specific accession numbers for the draft WGS data for each strain or plasmid are given in Table S1 (available as Supplementary data at *JAC-AMR* Online).

## Results

### Relationship between period of strain isolation and antimicrobial susceptibility

Among the 243 *N. gonorrhoeae* strains examined, 102 (42.0%) were resistant to penicillin, 34 (14.0%) to ciprofloxacin and one (0.4%) to spectinomycin, whereas seven (2.9%) were not susceptible to cefixime (Figure 2). No strain was non-susceptible or resistant to ceftriaxone or azithromycin. High-level ciprofloxacin-resistant (CIP-R^High^) strains (MIC ≥ 8 mg/L) were first identified after 2001, and strains not susceptible to cefixime were first isolated after 2001. By contrast, strains resistant to penicillin were primarily isolated in the 1980s, but decreased in the subsequent decade.

### MLST

MLST classified 243 strains into 83 distinct STs. Among these, the STs containing 10 or more strains included ST1901 (*n* = 17), ST1918 (*n* = 13), ST1594 (*n* = 12), ST1927 (*n* = 12), ST1963 (*n* = 12), ST1920 (*n* = 11), ST1590 (*n* = 10) and ST6959 (*n* = 10). ST1901 was initially identified in 2001, and its prevalence increased progressively thereafter (Figure 3). The genetic relationships between the ST variants are illustrated with a Grape Tree diagram (Figure S1). ST7363 and ST1596 were first detected in 2001, and have only maintained a relatively modest prevalence. Excluding ST1901, which boasts high prevalence, other STs detected before 2000 have been under continuous observation. The tally of different STs was 42 in 1971–1979, 34 in 1980–1989, 22 in 1990–1999 and 13 in 2001–2005 (no data for 2000 were available).

### *penA* allele and antimicrobial susceptibility

A total of 33 *penA* alleles were detected. Among them, *penA* alleles encoding PBP2 with a mosaic-like mutation (*penA*^mosaic^) were identified as 10.001 (*n* = 6) and 10.016 (*n* = 1). Strains carrying these *penA*^mosaic^ mutations showed a cefixime MIC of ≥0.25 mg/L (Figure 4a) and a ceftriaxone MIC of ≥ 0.125 (Figure 4b). Conversely, a subset of strains carrying *penA* alleles encoding PBP2 with non-mosaic-like (*penA*^non-mosaic^) mutations (*penA* type: 4.001, 5.002, 7.001, 12.001 and 13.001) also demonstrated a cefixime MIC of  ≥ 0.25 mg/L and ceftriaxone MIC of ≥ 0.125 (Figure 4a and 4b). Notably, seven strains carrying *penA* 10.001 or *penA* 10.016 were detected in or after 2001 (Table S1) and were classified as ST7363 or ST1596 (Figure 5).

### *penA* allele and MLST

Fourteen of the 33 *penA* alleles were detected in multiple strains across various STs (Figure 5). Conversely, *penA* 22.001 was found in 26 STs, *penA* 2.002 in 14 STs, *penA* 4.001 in 12 STs, *penA* 1.001 in seven STs and *penA* 12.001 in seven STs. *penA* 10.001 was identified in ST1596 (*n* = 3) and ST7363 (*n* = 3), whereas *penA* 10.016 was only identified in ST7363 (*n* = 1). Of the 83 STs detected, 22 carried various *penA* alleles (Figure 5).

### Number of mutations in QRDRs and susceptibility to ciprofloxacin

The range of ciprofloxacin MICs was ≤0.03–1 mg/L for strains with a single amino-acid substitution in a QRDR (*n* = 27), 8–64 mg/L for those with two substitutions (*n* = 22) and ≥64 mg/L for those with three substitutions (*n* = 6) (Figure 6). Despite having no mutation in a QRDR, two strains (TUM23515 and TUM23649) showed resistance to ciprofloxacin. Among the 28 strains with two or three substitutions, ST1901 (GyrA, S91F and D95G) was most common, containing 16 strains, whereas ST7363 (GyrA, S91F and D95N; and ParC, S88P) and ST1596 (GyrA, S91F and D95N; and ParC, S88P) contained three strains each (Figure 7).

### Period in which fluoroquinolone and ECS resistance determinants emerged

The first mutation detected in the QRDR during the study period was found in 1987 (Figure 8). The mutation was a single-residue change, either D95G or S91F, in the QRDR of GyrA. Double or triple mutations were first found in the QRDR in 2001. Strains carrying *penA* 10 were first identified in 2001 (Figure 8). All seven strains carrying *penA* 10 also had double or triple mutations in the QRDR.

### Identification of determinants of fluoroquinolone resistance other than mutations in the QRDR of *gyrA* and *parC*

We performed an additional analysis to identify the determinants of fluoroquinolone resistance beyond mutations in the QRDR of *gyrA* and *parC*. Our alignment of the available contig data from strain TUM23515, which showed ciprofloxacin resistance without QRDR mutations, and the WHO F reference genome yielded some findings. Although identifying determinants of fluoroquinolone resistance is challenging, we discovered two mutations in the region upstream from *norM* encoding the NorM efflux pump. The first mutation was the insertion of an ‘A’ nucleotide 84 bp upstream from *norM*, and the second was a ‘T’ nucleotide insertion 123 bp upstream from *norM*. These mutations may increase *norM* expression, although no further analysis to quantify the transcript or protein levels has been performed. A mutation of arginine to glutamine at position 652 (R652Q) in GyrB was also found in TUM23515, similar to that observed in the reference strains WHO L, WHO V and WHO Y, all of which show ciprofloxacin resistance.

### Identification of spectinomycin-resistance determinants

To identify the determinants of spectinomycin resistance, we aligned the nucleotide sequence of the 16S rRNA genes or 5S rRNA genes of TUM23498 and 14 WHO reference strains. A point mutation, C453T, was detected in the 16S rRNA gene of TUM23498. No other previously reported spectinomycin-resistance determinants, such as the mutation C1192T in the 16S rRNA gene or the deletion of codon 27 (valine) or the substitution of K28E in the ribosomal protein 5S, were observed.^34,35^

### Identification of tetracycline resistance determinants

We analysed the tetracycline resistance determinants in 35 strains for which draft WGS data were available, because the high-throughput typing method was not designed to target tetracycline resistance determinants. None of the strains tested positive for *tet(M)*. All strains, except TUM23498, showed an amino-acid substitution in the *rpsJ*-encoded protein that changed the valine at position 57 to methionine (V57M).

## Discussion

In this study, we retrospectively applied a high-throughput genotyping method and draft WGS to 243 *N. gonorrhoeae* strains isolated between 1971 and 2005 in Japan. Our goal was to understand the evolution of *N. gonorrhoeae* AMR within our region using molecular epidemiological typing. No strains belonging to STs that displayed reduced susceptibility to cefixime or CIP-R^High^ (ST1901, ST7363 and ST1596) were isolated before 2000. A decline in ST diversity in the gonococcal population closer to the present era suggests that these successful STs have become dominant. The increased isolation frequency of gonococci displaying resistance to both fluoroquinolones and ESCs is probably attributable to several novel lineages. The ancestral lineage acquired QRDR amino-acid substitutions or mosaic-like *penA*.

Strains carrying *penA*^mosaic^ (*penA* 10.001 and 10.016) demonstrated reduced susceptibility or resistance to ESCs, although a proportion of the strains with *pen*A^non-mosaic^ also displayed similar characteristics. The presence of amino-acid point mutations or other resistance mechanisms may contribute to this reduced susceptibility or resistance to ESCs. An increased number of amino-acid substitutions in QRDRs correlated with an increasing MIC for ciprofloxacin. We identified two strains (TUM23515 and TUM23649) lacking substitutions, which presented slightly reduced susceptibility to ciprofloxacin (MIC 1–2 mg/L), but the inherent resistance mechanisms remain unclear. TUM23515 contained two insertions upstream from *norM*, which may contribute to ciprofloxacin resistance by increasing the expression of *norM*. TUM23515 also contained a GyrB R652Q mutation outside the QRDR, which is unlikely to contribute to ciprofloxacin resistance.^34^ The baseline for the spectinomycin MIC was high, with one strain displaying resistance. Spectinomycin-resistant strains are uncommon at present, but several strains were isolated in the late 1980s.^35^ The point mutation C453T in the 16S rRNA gene could represent a novel spectinomycin-resistance determinant. This hypothesis can be confirmed by naturally transforming *N. gonorrhoeae* with the genomic DNA of the spectinomycin-resistant strain and then checking the transformant for spectinomycin resistance and sequencing its genome. The absence of azithromycin-resistant strains from 1972 to 2005 justifies the previous use of azithromycin monotherapy for gonorrhoea. However, prolonged exposure to azithromycin may have led to an accumulation of mutations in azithromycin-resistance determinants, resulting in the recent selection of resistant strains.^3^ Although tetracycline susceptibility testing was not conducted in this study, the presence of *tet(M)* and the *rpsJ* V57M codon mutation suggests that most strains were probably resistant to tetracycline, but not at a high level.

According to previous reports in Japan, the resistance rate of *N. gonorrhoeae* to ciprofloxacin has seen a significant rise, increasing from 6.6% in 1993–1994 to 24.4% in 1997–1998, 78.3% in 2009–2010 and 76.5% in 2016–2017.^10,36,37^ Regrettably, neither MLST nor AMR determinants were mentioned. Although the present study involved a smaller number of strains per year than its predecessors, it fills the gaps in molecular epidemiological data. We found that CIP-R^High^ ST1901 strains carrying *penA*^non-mosaic^ (*penA* 5.002 or *penA* 13.001) could be ancestral to CIP-R^High^ ST1901 carrying *penA*^mosaic^ (*penA* 10.001-like or *penA* 34.001-like), isolated between 1998 and 2005 in Japan. ST1901 appears to have initially acquired high-level ciprofloxacin resistance, followed by ESCs resistance.^13,19^ The ST1596 and ST7363 strains we discovered had already acquired CIP-R^High^ and *penA*^mosaic^ (*penA* 10.001 or *penA* 10.016). ST1596 and ST7363 acquired CIP-R^High^ and *penA*^mosaic^ earlier than ST1901, but ST1901 has since predominated over the other STs.

This study had four possible limitations. First, the strains isolated between 1971 and 2005 were randomly selected, with a cap of 12 each year. This sample size may be inadequate for a comprehensive analysis of the diversity of STs within the *N. gonorrhoeae* population in Japan. Furthermore, we acknowledge a shortage of strains in certain years. However, we determined that the ST diversity was lower in the strains isolated during the 1990s and 2000s than those isolated between in 1970s and 1980s. Second, the strains were sourced from a restricted range of locations in Japan, with Tokyo and Kanagawa Prefecture the primary locations. These had the largest and third-largest populations in Japan between 1971 and 2005, respectively. Third, the genetic relationships within each ST could not be fully determined because most of these strains were analysed with a high-throughput genotyping method. Fourth, a transformation analysis was not conducted to identify the AMR determinants contributing to the AMR phenotype.

In this study, we have demonstrated a significant reduction in the diversity of *N. gonorrhoeae* STs from 1971 to 2005. We identified the STs of the CIP-R^high^ and CFM-R that newly emerged during that period. We also identified ST1901 as the probable ancestor of CIP-R^high^ and CFM-R, which would later acquire *penA*^mosaic^.

## Supplementary data

Figure S1 and Table S1 are available as Supplementary data at *JAC-AMR* Online.
