# Supplementary figures and images for: Molecular epidemiological and antimicrobial-resistant mechanisms analysis of prolonged Neisseria gonorrhoeae collection between 1971 and 2005 in Japan
Source: JAC Antimicrob Resist. 2024 Mar 12;6(2):dlae040. doi: 10.1093/jacamr/dlae040 (PMC10928670; doi:10.1093/jacamr/dlae040)

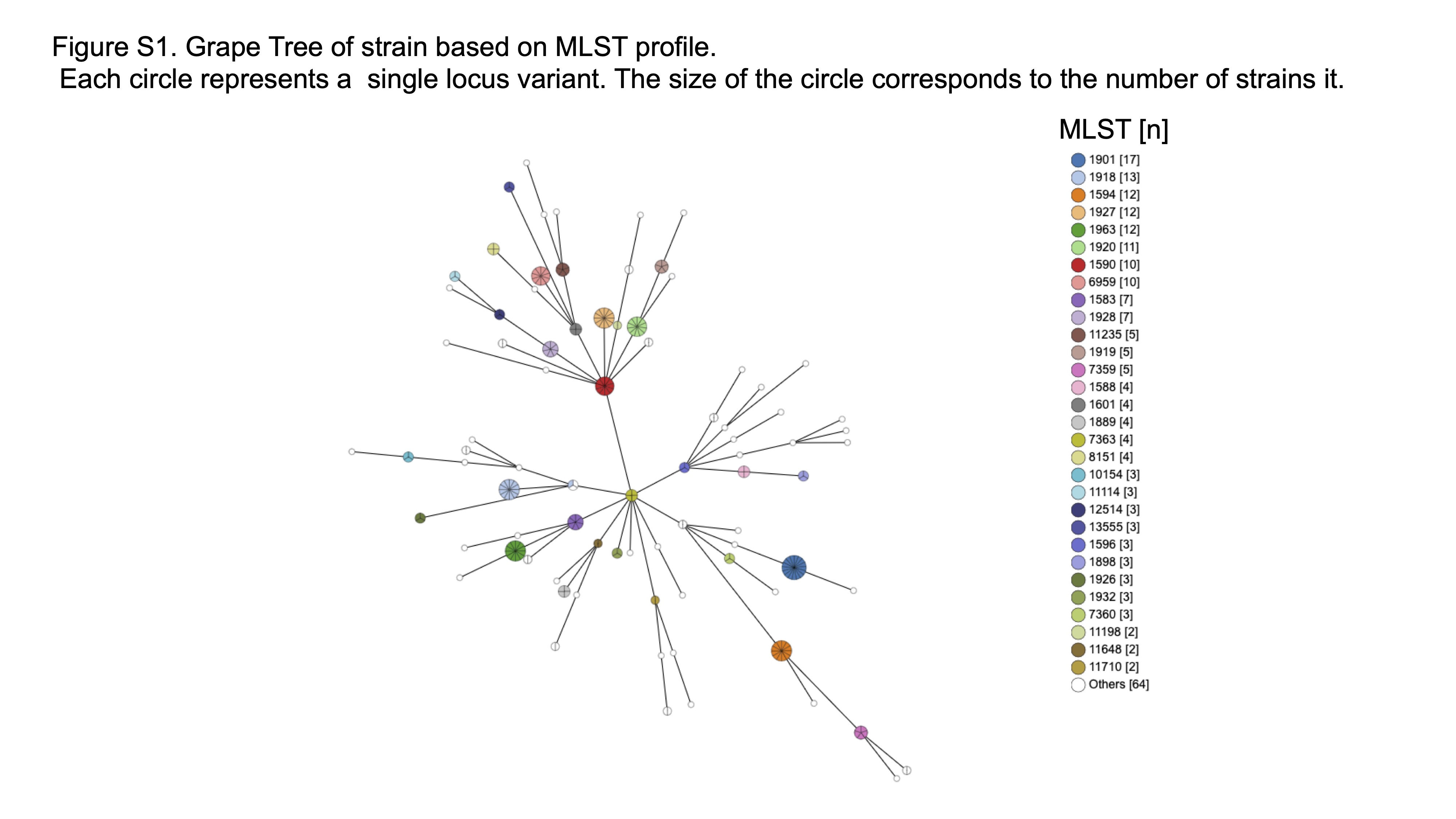

Supplement: dlae040_Supplementary_Data [file dlae040_supplementary_data.zip › Ngono_Kanagawa_Figure S1_v1.jpg]
